# A Rare Case Report of Mesenchymal Chondrosarcoma with Pancreatic Metastasis

**DOI:** 10.3390/medicina58050639

**Published:** 2022-05-05

**Authors:** Jian-Jiun Chen, Cheng-Wei Chou

**Affiliations:** 1 Division of Hematology/Medical Oncology, Department of Internal Medicine, Taichung Veterans General Hospital, Taichung 40705, Taiwan; jianjiunchen@gmail.com; 2 Graduate Institute of Biomedical Sciences, China Medical University, Taichung 404333, Taiwan

**Keywords:** mesenchymal chondrosarcoma, pancreatic metastasis, extra-skeletal mesenchymal chondrosarcoma

## Abstract

*Background*: Mesenchymal chondrosarcoma is a rare but aggressive subtype of sarcoma. The majority of involvement locates in the axial skeleton. Treatment modalities include radical surgery, local radiotherapy, and systemic chemotherapy. However, the long-term survival outcome remains poor. *Case presentation*: We present the case of a 33-year-old male with a palpable chest wall mass for one year, diagnosed with mesenchymal chondrosarcoma with surgical removal. Later, he had an unusual pancreatic tail tumor as the first presentation of disease metastasis which was proven by surgical resection one year later. *Conclusion*: Although mesenchymal chondrosarcoma locates mainly in the axial skeletal system, extra-skeletal soft tissue or organ involvement might be seen occasionally. Active surveillance with multidisciplinary team management could significantly prolong survival outcomes.

## 1. Introduction

Mesenchymal chondrosarcoma (MC) was first described by Lightenstein and Bernstein in 1959 [1]. Less than 3% of primary chondrosarcomas are mesenchymal chondrosarcomas, usually with a high histological grade. The most common primary sites are the craniofacial bones, the vertebrae, the ribs, the ilium, and the femur [2]. Visceral organ involvement is rare. Few sporadic extra-skeletal mesenchymal chondrosarcomas (ESMCs) have been reported, mainly in the head, neck, thorax, abdomen, and retroperitoneum [3]. Men and women have equal incidence rates. The highest incidence is in the second and third decades of life [2]. Due to the few cases published until now [4], we present a case of mesenchymal chondrosarcoma with unusual distant pancreatic metastasis as a recurrent presentation.

## 2. Case Presentation

A 33-year-old male presented with a painless, palpable chest wall mass for about one year with a gradually increased size. The breast sonography showed a 7.5 cm oval mass with heterogeneous echogenicity and calcifications. He received an image-guided biopsy with morphology showing sheets of primitive round and oval cells with foci of cartilaginous differentiation and abrupt transitions with surrounding round cells. The immunohistochemistry stain showed AE1/AE3 (−), LCA (−), actin (−), desmin (−), S100 (+ on the chondroid island), CD34 (−), CD99 (+), TLE-1 (+), and FLI-1 (−). There was no available gene fusion result. Based on the morphology and immunohistochemistry stain results, mesenchymal chondrosarcoma was diagnosed. Without evidence of distal metastasis, he received chest wall excision with partial resection of the left third, fourth, and fifth ribs and reconstruction with a left latissimus dorsi muscular flap. The pathologic stage revealed T2N0M0, FNCLCC grade 3. The pathology also showed a deep surgical margin involved. He refused adjuvant local radiotherapy or chemotherapy. Then, he received active surveillance after surgery without evidence of tumor recurrence for about one year. 

**Figure 1:**
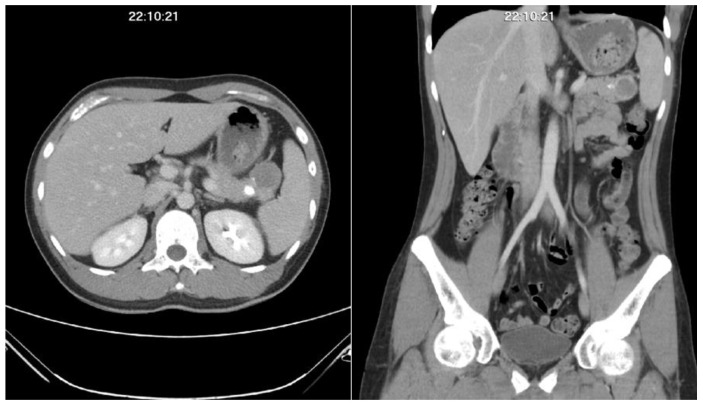
Abdominal computerized tomography scan result. The patient had a poorly enhanced mass lesion with calcification over the pancreatic tail region found during the follow-up computerized tomography scan one year after the initial diagnosis.

**Figure 2:**
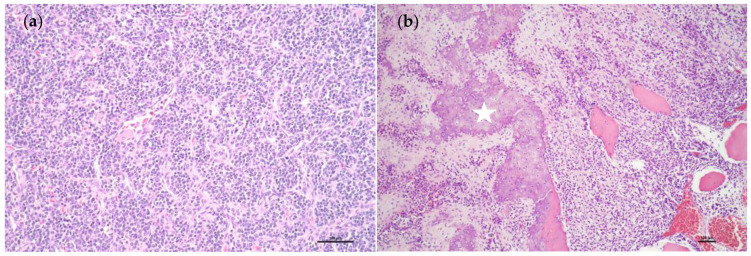
The immunohistochemistry stain of the pancreatic mass. The histological result revealed small blue round and spindle-shaped mesenchymal cells ((**a**), 200×). In addition, the picture shows the islets of highly differentiated cartilage (indicated by a white star) transited abruptly from the surrounding blue round cells ((**b**), 100×).

During the regular follow-up abdominal computerized tomography scan one year after the diagnosis, a new poorly enhanced mass lesion about 3.4 cm in size with calcified nodules was detected over the pancreatic tail (Figure 1). He received subsequent laparoscopic pancreas distal partial pancreatectomy for the rare presentation and tissue proof. The surgical pathology result confirmed the metastatic mesenchymal chondrosarcoma of the pancreas (Figure 2). 

Six months later, he further developed tumor recurrence over the scalp and left axilla, and superior sagittal sinus involvement. He successfully underwent occipital craniectomy with tumor removal and cranioplasty successfully without significant neurological deficits. However, the disease progressed four months later with several metastatic lesions, including on the ribs, cervical spine, femoral neck, pelvis, and lymphadenopathies. Then, he received palliative chemotherapy with a MAID regimen (mesna 2000 mg/m^2^/day, Adriamycin 20 mg/m^2^/day, ifosfamide 2000 mg/m^2^/day, and dacarbazine 250 mg/m^2^/day as a continuous infusion over 72 h) for six cycles. The follow-up computerized tomography scan showed stable disease, and he still received systemic chemotherapy for further disease control.

## 3. Discussion

Mesenchymal chondrosarcomas are highly malignant tumors. They are characterized by differentiated cartilage admixed with solid, highly cellular areas composed of undifferentiated small round cells [5]. The exact pathogenetic mechanisms behind mesenchymal chondrosarcoma are still unknown. However, some studies observed that chromosome 8 might be related to mesenchymal chondrosarcoma tumorigenesis [6].

The average age is 25 to 30 years [7], younger than other subtypes of chondrosarcoma. A high proportion of extra-skeletal primary tumors are not seen with other chondrosarcoma subtypes [8]. Of the approximately one third of cases that affect the extra-skeletal soft tissues, the meninges are one of the most common sites [9]. In contrast to conventional chondrosarcomas, mesenchymal tumors most commonly involve the axial skeleton, including the craniofacial bones (especially the jaw) [10], ribs, ilium, and vertebra. Approximately 20 percent of cases have metastatic disease at diagnosis [11]. The overall survival rate of mesenchymal chondrosarcoma is 51% and 43% at 5 and 10 years, respectively. No difference in overall survival has been detected between extra-skeletal and skeletal tumors. 

Most importantly, the presence of tumor metastasis and a 1 cm size increase were both independently associated with an increased risk of death [12]. Another earlier study reported that mesenchymal chondrosarcoma’s five- and ten-year survival rates were 54.6% and 27.3%, respectively [2]. As a result, the prognosis for patients with mesenchymal chondrosarcoma is usually poor, and long-term follow-up is necessary.

Clinically, patients with pancreatic metastases usually present with abdominal pain and icterus, while some remain asymptomatic and are diagnosed on routine follow-up [13]. Radiologically, a computed tomography scan or a magnetic resonance image is advised for diagnosis [3]. The computed tomography scan shows granular irregular calcifications with a surrounding hypodense tumor. Meanwhile, the magnetic resonance image characteristically shows low-intensity calcified areas surrounded by a high-intensity tumor on T2-weighted images, suggesting metastasis [14]. However, compared with conventional chondrosarcoma, the image of mesenchymal chondrosarcoma is not specific. In addition, fine-needle aspiration of the pancreas has been used more frequently to diagnose pancreatic masses [13].

Histologically, the tumor is characterized by poorly differentiated small round cells with an abrupt transition to hyaline cartilage [3]. The immunohistochemical stains are usually positive for NKX2.2, CD99, S100, and SOX9 [15]. Recently, SOX-9 has been reported as a marker that stains both the undifferentiated and the cartilaginous components in ESMC [16,17].

Due to the limited cases and lack of large-sized randomized clinical trials, mesenchymal chondrosarcoma has no standard treatment. However, based on several case experiences, the consensus is that patients should receive radical surgery and possible chemotherapy [4]. The best chemotherapy regimen is not well established. Based on currently available results, either a Ewing sarcoma-based multi-drug regimen or osteosarcoma-type doxorubicin plus cisplatin-based chemotherapy regimen may be used [18,19]. Neoadjuvant therapy may be considered if the tumor status is at the non-metastatic stage. Moreover, radiotherapy for the therapeutic approach has been reported [11]. The use of radiotherapy might also improve local control in those cases with local disease [20].

In the presented case, an unusual presentation with a pancreatic tail mass as the first distal metastasis was detected one year after the initial diagnosis. We arranged a laparoscopic pancreas distal partial pancreatectomy for surgical resection. The histologic examination confirmed a metastatic mesenchymal chondrosarcoma. Further, the patient received palliative chemotherapy with the MAID regimen with the best stable disease response and still received treatment. We summarized the cases with an unusual metastatic site of the pancreas from the literature. A total of 16 cases have been reported (Table 1). There were 9 females of a total of 16 patients, without noticeable gender differences. The average diagnosed age was 35 years old, corresponding to the epidemiology result [2]. A total of 13 cases were reported as secondary mesenchymal chondrosarcoma of the pancreas. Only two patients were diagnosed with a primary tumor, and one had no reported result. The majority of treatment strategies were surgery with further systemic chemotherapy. 

## 4. Conclusions

Mesenchymal chondrosarcoma is a rare but poor-prognosis tumor. Metastasis and tumor size contribute to decreased survival outcomes. Thus, long-term follow up is necessary, and earlier diagnosis and treatment are indispensable. In addition, although mesenchymal chondrosarcoma is usually located in the skeletal system, an extra-skeletal site is also possible. A metastatic tumor should be considered when presented with a pancreatic mass in mesenchymal chondrosarcoma patients. Diagnostic tools include image studies such as computerized tomography scans and magnetic resonance images. An immunohistochemistry stain reviewed by an experienced pathologist is warranted. Treating metastatic mesenchymal chondrosarcoma with radical surgery with chemotherapy might improve the long-term outcome.

## Figures and Tables

**Table 1:** Literature review of published case reports with pancreatic metastasis.

Author	Year	Gender	Age	Primary or Secondary	Size (cm) and Location (Head/Body/Tail)	Treatment	Outcome *	Primary Site	Metastatic Site	Latency Period for Pancreatic Metastasis (y)
Byun et al. [21]	1995	Female	36	Secondary	7.7 × 4.3 × 5/Tail	Distal pancreatectomy, CT	NR	Thigh	Pancreas	Synchronous
Komatsu et al. [22]	1999	Female	28	Secondary	2.5/Tail	Distal pancreatectomy	NR	Meninges	Pancreas	17
Yamamoto et al. [23]	2001	Male	29	Secondary	NR/Body, Tail	Distal pancreatectomy and enucleation of the head of the pancreatic tumor	>120	Thigh	Pancreas, lung, testis, skin, chest wall	3
Naumann et al. [24]	2002	Female	24	Secondary	NR/NR	RT, CT	>84 **	Retroperitoneum	Kidney, lung, rib, humerus, pancreas, spine	6
Trembath et al. [25]	2003	Female	27	Secondary	9.5/NR	CT, partial pancreatectomy	NR	Tibial	Retroperitoneum, pancreas, diaphragm	2
Chatzipantelis et al. [26]	2006	Male	26	Secondary	3.8 × 3.5/Tail	Distal pancreatectomy	NR	Brain	Lung, thigh, pancreas	9
Oh et al. [27]	2007	Male	41	Primary	13 × 12 × 7/Body, Tail	Enucleation	NR	Pancreas	-	-
Bu et al. [16]	2010	Female	34	Primary	18 × 16/Body, Tail	Surgical resection of pancreas body and tail	>52	Pancreas	-	-
Tsukamoto et al. [28]	2014	Male	39	Secondary	5 × 6/Body, Tail	Distal pancreatectomy, CT	34	Buttocks	Pancreas, sacrum, ilium, ischium, lungs	Synchronous
Smith et al. [13]	2015	Female	44	Secondary	NR/Body	Distal pancreatectomy, CT	>24	Chest wall	Pancreas (recurrence)	21
Guo et al. [29]	2015	Male	40	Secondary	2 × 3 × 2/Body	Distal pancreatectomy	>108	Femoral vein	Pancreas, lung, pleura, mediastinal and axillary lymph nodes	3
Cohen et al. [30]	2016	Female	32	Secondary	2.9/Tail	Laparoscopic distal pancreatectomy, CT	15	Pterygoid region	pancreas, lung	8
Cohen et al. [30]	2016	Female	38	Unknown	9.5/Tail	Distal pancreatectomy, neoadjuvant CT	>40	Unknown	lung, pancreas, ilium, femur	Synchronous
Shah et al. [14]	2019	Male	49	Secondary	5/Tail	Distal pancreatectomy	>11	Thigh	Pancreas	10
Camacho et al. [31]	2020	Female	53	Secondary	3/Tail	Laparoscopic distal pancreatectomy	>24	Lower limb	Lung, pancreas	7
Sun, J. et al. [32]	2021	Male	21	Secondary	4.94/Neck	NR	NR	Rib	Pancreas, adrenal gland	Synchronous
Present case	2021	Male	34	Secondary	3.4/Tail	Distal pancreatectomy, CT	>3	Chest wall	Pancreas	1

* Outcome: months of survival after surgery. ** Months of survival after diagnosis of MC. CT: chemotherapy; RT: radiotherapy; NR: not recorded; SSS: superior sagittal sinus.
